# Strategies for swimming: explorations of the behaviour of a neuro-musculo-mechanical model of the lamprey

**DOI:** 10.1242/bio.20149621

**Published:** 2015-02-06

**Authors:** Thelma L. Williams, Tyler McMillen

**Affiliations:** 1 Department of Mechanical and Aerospace Engineering, Princeton University, Princeton, NJ 08544, USA; 2 Department of Mathematics, California State University at Fullerton, Fullerton, CA 92834, USA; * Present address: 17 Carr Road, Walkley, Sheffield, S6 2WY, UK.

**Keywords:** Fish, Swimming, Fluid dynamics, Locomotion, Muscle

## Abstract

Experiments were performed on a neuro-musculo-mechanical model of a lamprey, to explore the strategies for controlling swimming speed. The muscle component of the model was based on previous experiments on isolated lamprey muscle. The patterns of muscle activation were those found in EMG studies on swimming lampreys. The fluid mechanics were modelled with G.I. Taylor's simplification. Tail beat frequencies of 2–6 sec^−1^ were combined with muscle activation strengths of 0.1% to 20% of maximum tetanic isometric strength. The resulting forward swimming speed and changing body shape were recorded. From the changing body shape the speed of the backward-travelling wave of curvature was calculated, as well as the ratio between the speeds of the waves of activation and curvature. For any given activation strength there was a tail beat frequency that gave maximal forward speed. Furthermore, for all the combinations of activation strength and tail beat frequency that gave such maximum swimming speeds, the ratio of the speed of the wave of curvature to the wave of muscle activation was approximately 0.75. This is similar to the ratio found in swimming lampreys.

## INTRODUCTION

Fish swim by generating waves of muscle activation that pass down the body toward the tail. Such activation produces travelling waves of lateral curvature, which develop forward thrust from the surrounding water. Increased swimming speed can be brought about by increasing either the frequency of the waves or the strength of muscle activation or both.

One of the interests of this study is to investigate the effects on swimming speed of increasing each of these qualities, in the hope of gaining insight into the way it is done in the real animal. We will look for patterns that could give insight into the strategies of swimming. For example, we will measure the swimming speed at different frequencies for a given level of muscle activation, and at different levels of activation for a given frequency. We will then try to interpret the results.

Grillner and Kashin (Grillner and Kashin, 1976) first showed that the waves of muscle activation travel down the body of an eel faster than the resulting waves of body curvature. Williams et al. (Williams et al., 1989) quantified this relationship for the lamprey, showing that the relationship between the speed of the two waves remains statistically independent of the swimming speed. Since then, this feature has been demonstrated for steady swimming in every fish species in which it has been investigated (Gillis, 1998; Altringham and Ellerby, 1999).

Because of this mismatch of wave speeds, the swimming muscles are active during muscle lengthening for an increasing fraction of the cycle as the waves travel toward the tail. Positive work cannot be done by muscle if it is being lengthened while producing force, so the observed mismatch between activation and curvature at first seemed puzzling. Blight (Blight, 1976) suggested that such timing causes an increased stiffness of the tail as it moves laterally, enabling it to better oppose the reactive force of the water. This hypothesis was developed further by Long and Nipper (Long and Nipper, 1996). The main goal of this study is to develop and test hypotheses about the possible advantage to the swimming animal of this mismatch of activation and curvature.

There are no systematic kinetic studies on free-swimming lampreys, such as there are on many species. This is because lampreys do not seem capable of being trained to swim regularly in captivity (personal observation). When not swimming to catch prey or to travel upstream to spawn, a lamprey generally attaches by its sucker to a prey fish and is carried along. In captivity they typically attach to the sides of the tank in which they are confined. When disturbed they swim briefly before re-attaching elsewhere. Studies conducted in a swim-mill have been limited and have required tethering and being held in the flowing stream. For this reason we have chosen to conduct studies on a neuro-musculo-mechanical model of a free-swimming lamprey. In addition, independent control of variables such as frequency of swimming and strength of muscle activation is possible, as it is not in an intact animal.

There have been four major computational models of anguilliform swimming to date. Carling et al. (Carling et al., 1998) presented the first model of a self-propelled swimmer. The changes in body shape were specified at the start and the simultaneous solution of the Navier-Stokes equations of fluid flow and Newton's equations of motion of the body produced swimming of the model creature through the surrounding fluid. This model suffered from being only two-dimensional. Furthermore, the resulting patterns of water flow did not resemble those of an anguilliform swimmer, as shown in data by Tytell and Lauder (Tytell and Lauder, 2004). Unfortunately, a fault in the computation has since been discovered by the authors (T.W., unpublished). Kern and Koumoutsakos (Kern and Koumoutsakos, 2006) performed a fully three-dimensional Navier-Stokes computation for anguilliform swimming in which the changing body shape was determined by an algorithm which optimized either the swimming efficiency or the burst swimming speed. The patterns of water flow were similar to those seen in swimming eels (Tytell and Lauder, 2004). McMillen et al. (McMillen et al., 2008) published the first computation including a realistic model of muscle physiology, such that the changing body shape was produced by muscle activation within a mechanical model of the body interacting with fluid forces. The fluid mechanics computation was simplified according to Taylor's resistive model (Taylor, 1952), which does not model vortex flow. Tytell, Hsu and others (Tytell et al., 2010) solved the full Navier-Stokes equations, using the immersed boundary method (Peskin, 2002), but only in two dimensions and with a simplified model of muscle force production.

We have chosen to use the model of McMillen et al. (McMillen et al., 2008), but with a more realistic body shape and a more advanced model of muscle force generation. This muscle model includes the phenomenon of work-dependent deactivation (WDD) (Josephson, 1999), and thereby produces a more accurate prediction of responses of isolated muscle preparations to stimulation during sinusoidal movement (Williams, 2010). This also allows the use of higher frequencies of activation than in McMillen et al. (McMillen et al., 2008).

We believe this model comes closest to being based on Newton's laws of motion and the physiological properties of muscle. In due course we intend to expand the model to use the full Navier-Stokes equations.

## RESULTS I

**Fig. 1.**
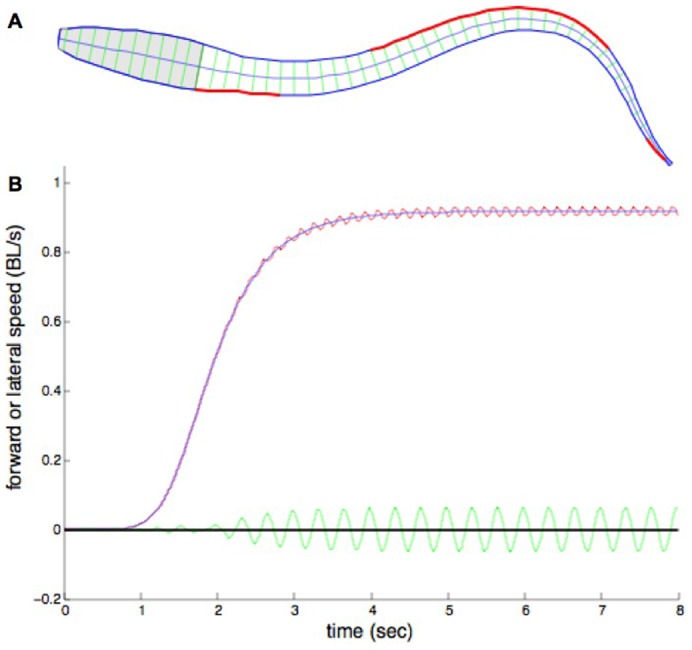
Typical experiment. (A) 50-segment body cross section, from above. Centre line represents notochord. Each segment has swimming muscle connecting the cross bars (green) on each side. Shaded section represents head and gills, in which there is no swimming muscle. Red lines represent hemi-segments in which there is active muscle at this time. Those sections of the body without red lines represent those where muscle is relaxing. (B) Time course of velocity of centre of mass during an experiment at activation frequency 3 s^−1^ and muscle activation strength 1%. Red line: forward velocity; blue line: forward velocity averaged over one half cycle; green line: lateral velocity.

In all experiments, the forward swimming speed oscillates with a frequency of two per swimming cycle, reflecting the travelling wave of curvature alternating on the two sides of the body (Fig. 1B). The lateral speed oscillates at a frequency of one per cycle, alternating to the left and the right. The mean forward swimming speed rises to a maximum as a steady-state is reached.

**Fig. 2.**
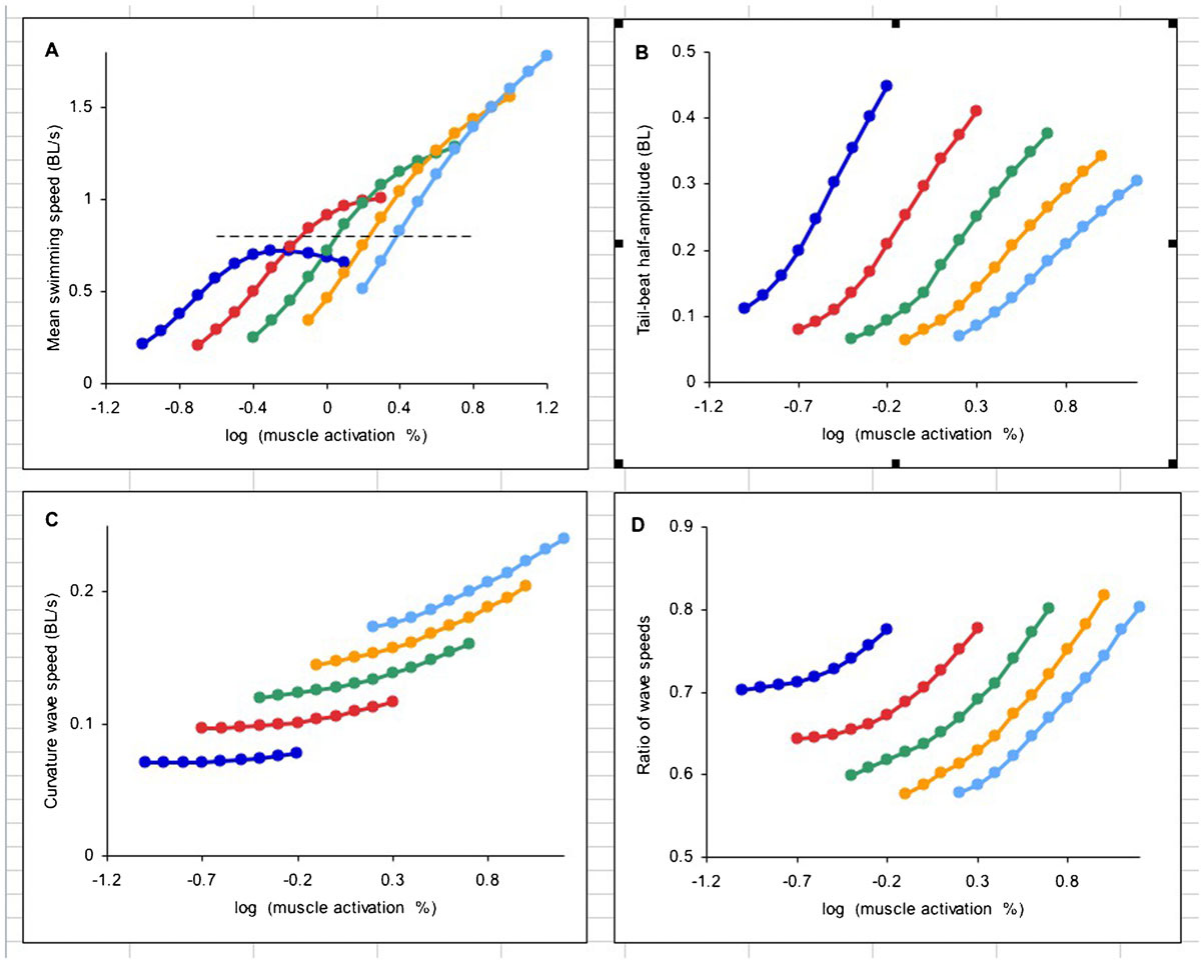
Results of simulations at a range of frequencies from 2 (dark blue) to 6 (pale blue), plotted against the logarithm to the base 10 of the level of muscle activation as % isometric tetanic maximum. (A) Mean forward swimming speed at steady-state. Dashed line at speed of 0.8 *BL*sec^1^. (B) Tail beat amplitude from midline to either side. (C) Speed of travel of wave of lateral curvature. (D) Ratio of curvature and activation wave speeds. At each frequency, the activation wave speed is constant.

At a given frequency, swimming speed increases with increasing muscle activation (Fig. 2A) until it reaches a maximum, after which it declines (shown only for slowest frequency). As muscle activation increases, so does the tail-beat amplitude (Fig. 2B). The speed of travel of the activation wave in body lengths (*BL*) per second is set by the frequency, equalling 1 *BL*/cycle times frequency in cycle s^−1^. The speed of travel of the curvature wave depends on the level of muscle activation as well as the frequency (Fig. 2C), such that the ratio of the wave speeds is not constant (Fig. 2D).

Except at the highest swimming speeds, a given forward speed can be achieved by a range of cycle frequencies (Fig. 2A). The dashed line, for example, shows that a speed of 0.8 *BL*/s can be achieved at four of the frequencies investigated, by the choice of appropriate activation strengths. The question arises as to how the particular combination of frequency and level of activation for a given forward speed is arrived at in the swimming animal.

**Fig. 3.**
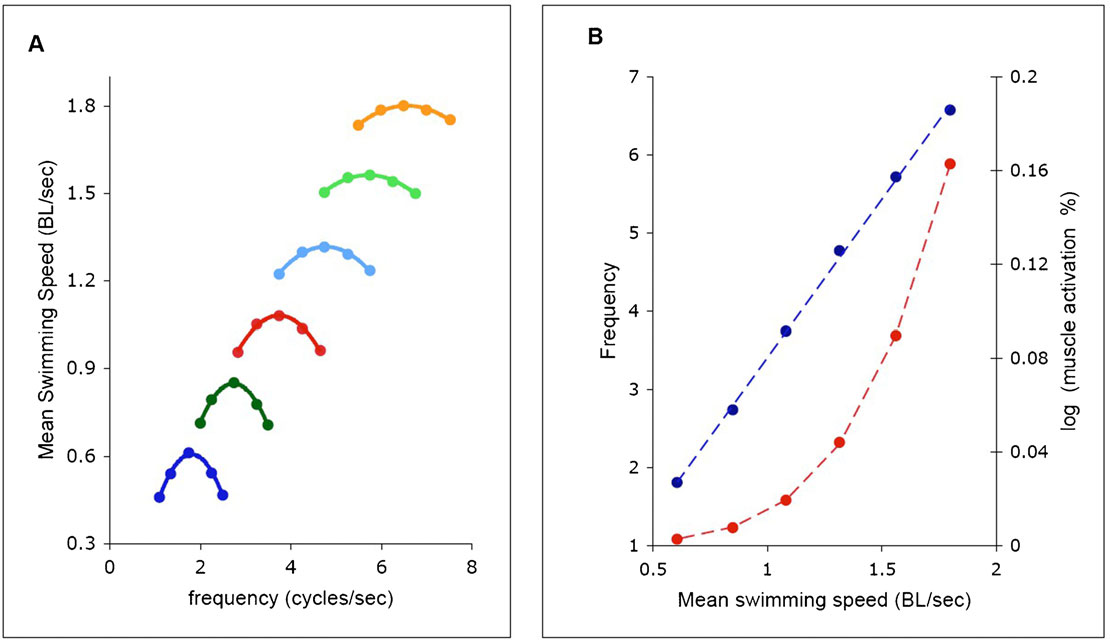
Maximal swimming speeds. (A) Mean forward swimming speed at a range of muscle activation strengths, from 0.3% (dark blue) to 18% (orange), plotted against cycle frequency. Lines are drawn from quadratic fit to data; *R*^2^ was greater than 0.99 in each case. (B) Values of frequency and muscle activation strength which gave maximal forward speed for each value of activation strength, taken from quadratic fits to data of A. Blue symbols: frequency; dashed line: linear regression *y* = 4.05*x*−0.642, *R*^2^ = 0.999. Red symbols: activation strength; dashed line 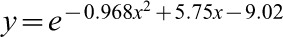, *p*<.001 (chi-squared test).

This was investigated by plotting swimming speed against cycle frequency at a range of muscle activation values (Fig. 3A). For a given level of muscle activation there exists a frequency at which the maximum speed is attained. The value of this frequency was determined in each case by fitting a quadratic equation (solid lines, Fig. 3A) and determining the frequency at which the maximum swimming speed would have occurred.

Fig. 3B plots the frequencies and muscle activation values taken from the maximum value of each curve in Fig. 3A. The frequency depends linearly on the swimming speed (upper curve, Fig. 3B). The activation (lower) curve was fitted by the exponential of a quadratic function of the speed. This equation was an empirical choice, as it gave a better fit than any other simple equation with 3 parameters. For fitting equations and statistics, see legend of Fig. 3.

## RESULTS II

### Comprehensive model

**Fig. 4.**
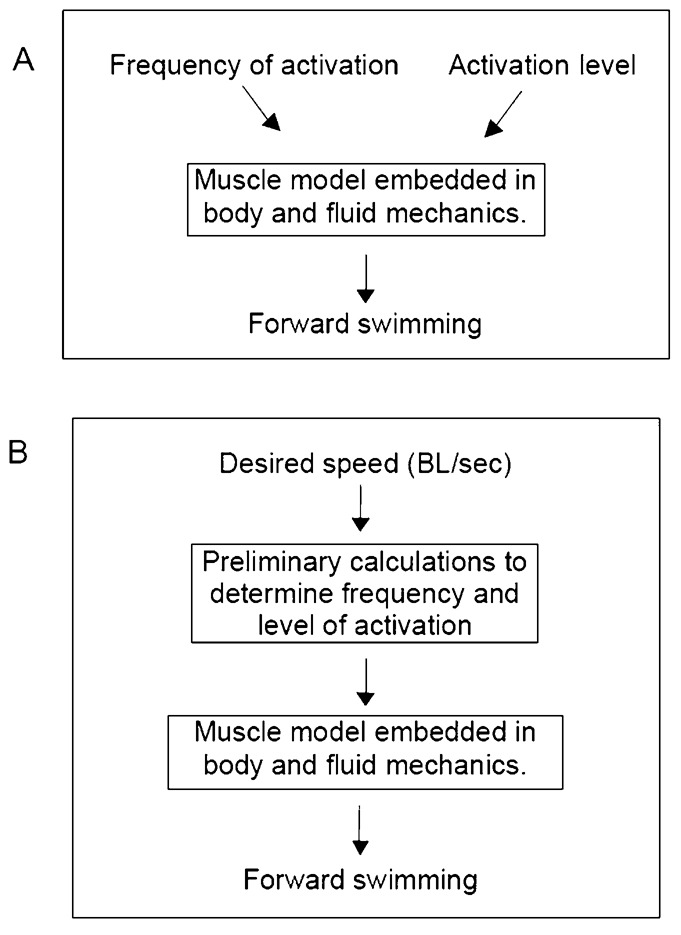
Diagram indicating form of computation. (A) Original scheme, where frequency and activation level are independent inputs to the computation, which gives rise to the data of Fig. 2 and Fig. 3A. (B) Scheme based on the data of Fig. 3B. Sole input is desired swimming speed, and output is forward swimming of the model. Results shown in Fig. 5.

These results suggest that one strategy used by the spinal cord for matching forward swimming speed to frequency and strength of activation could be to maximise the forward speed for a given level of muscle activation. To investigate this hypothesis further, we incorporated the equations of Fig. 3 into the full computation. The only input to the code was the desired swimming speed (see Fig. 4B), and the frequency and activation strength were calculated within the computation, according to the equations in the legend of Fig. 3B. In each case, the resulting forward swimming speeds differed from the input value by less than 1%.

**Fig. 5.**
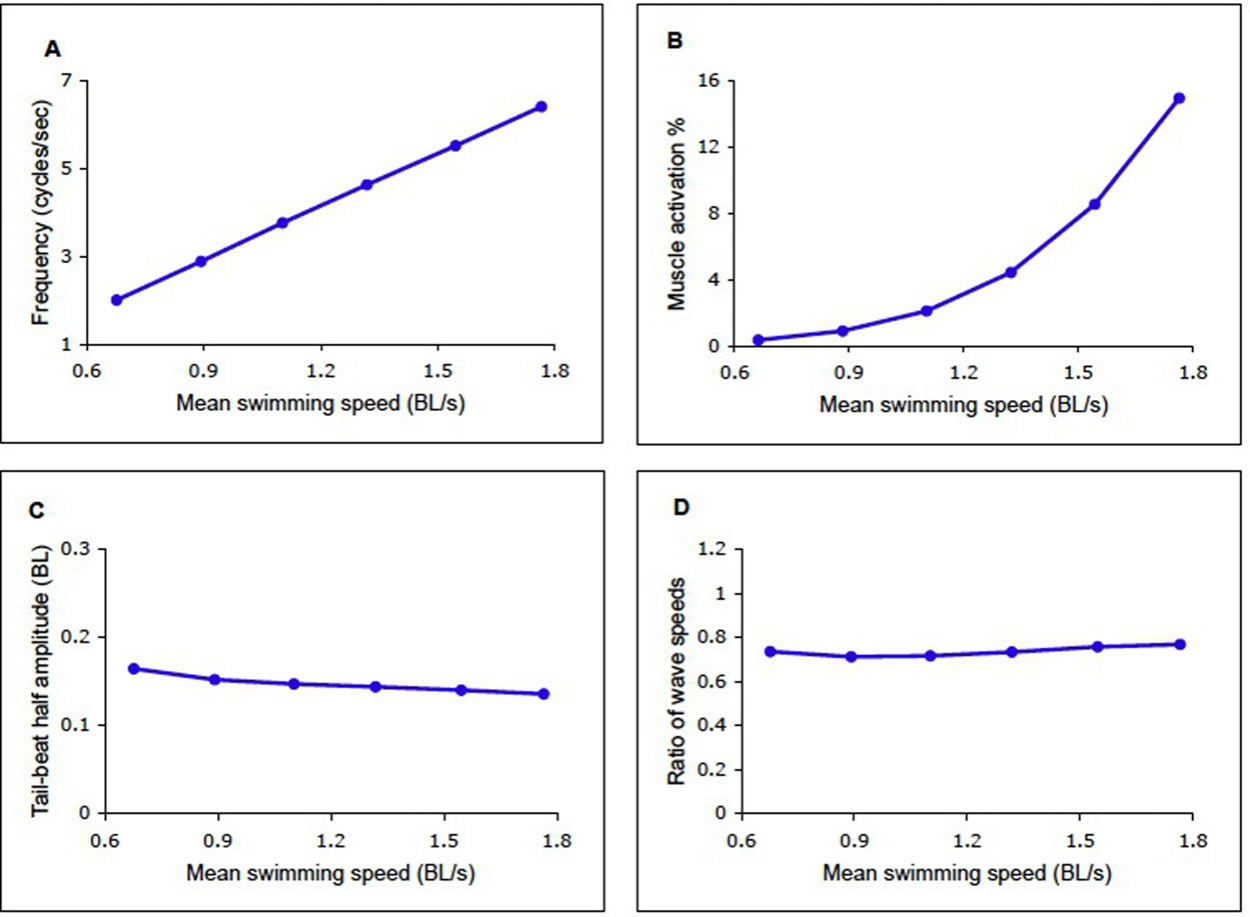
Results of computation carried out as in Fig. 4, using equations given in legend of Fig. 3. (A) Tail-beat frequency. (B) Muscle activation level. (C) Lateral excursion of the tail, from the midline to either side, in body lengths. (D) Ratio of the wave speeds of activation and curvature. Abscissa: input values (forward swimming speed) for the computations.

The results of this process are shown in Fig. 5, where each swimming speed is associated with a unique frequency and activation level, as in Fig. 3B. Swimming speeds between 0.6 and 1.8 *BL*/sec were used, encompassing the values in the experiments of Fig. 3B. This gave rise to frequencies between 2 and 6.4 cycle s^−1^ and activation levels between 0.3% and 1.6%. In Fig. 5A the linear relationship between frequency and swimming speed is shown, as in Fig. 3B, but with ordinate and abscissa swapped. Fig. 5B shows the swimming speed as a function of muscle activation, for the same six experiments. The tail excursion (Fig. 5C) and the ratio of the curvature and activation waves (Fig. 5D) are nearly constant.

The mean value of the wave travel ratio in Fig. 5D is 0.75, which is within the range observed in swimming lampreys of 0.72±0.07 (SD) (Williams et al., 1989). Within this study, each frequency can give rise to a range of values for this ratio, by the choice of values of muscle activation (Fig. 2D). Yet the combinations giving maximum speed in Fig. 3A gave ratios near 0.75. Thus it can be surmised that the value of this ratio is that which provides the greatest forward speed at any level of muscle activation.

## DISCUSSION

### Comparison with studies on swimming fish

The linear dependence of swimming speed on tail beat frequency was first shown by Bainbridge (Bainbridge, 1958), who found that the gradient for three species of fish that swim primarily with the action of their tails (dace, trout and goldfish) was 0.75 *BL*/cycle. Similar kinematic studies have not been made on lampreys, but studies on another angulliform swimmer, the eel, have also shown a linear relationship between frequency and swimming speed, but with a slope of approximately 0.41 *BL*/cycle (Gillis, 1998). This value is considerably lower than Bainbridge's tail-fin powered fish. The value of the gradient of 0.23 found here in the lamprey model (Fig. 5A) is even smaller. The lamprey is a more primitive species than the eel and its swimming less streamlined. It might thus be expected to swim more slowly at a given frequency.

Bainbridge (Bainbridge, 1958) found that in the same species as above, tail beat amplitude rises with increasing frequency to a maximum of about 0.1 *BL*, measured from the midline to the maximum on either side. In the eel, however, the tail beat amplitude does not vary even at lower frequencies, remaining at about .08 *BL* (Gillis, 1998). In the lamprey model the excursion was also approximately constant, at about 0.13 *BL* (Fig. 5C). Although there has not been a systematic measurement of this variable in swimming lampreys, published midlines of a swimming lamprey (Fig. 3F; Bowtell and Williams, 1991) indicate that the tail beat amplitude is greater than that seen in either the eel or the fish in Bainbridge's study B58.

### General conclusion

In a swimming animal, a given swimming speed is accomplished by a particular tail-beat frequency (Bainbridge, 1958), which will correspond to a particular level of muscle activation. The cycle frequency of the CPG determines the tail-beat frequency, and the intensity of each segmental burst determines the level of muscle activation. The present study indicates one way in which these values may be determined, by a process which matches cycle frequency with a given level of activation, such that speed is maximised (as opposed, for example, to efficiency). This strategy could be built implicitly into the spinal cord circuits comprising the central pattern generator (CPG). It is known that feedback is not required to set the intersegmental timing, since the intersegmental delay, i.e. the speed of travel of the activation wave, is the same in the spinal cord *in vitro* as in intact swimming animal (Wallén and Williams, 1984). Although that study also found no difference in the activation wave between intact and high spinal animals, the delay between activation and curvature could not be determined, since the animals were not filmed. Only electromyogram or electroneurogram measurements were made.

Although the speed of the wave of activation in *BL*/cycle does not require feedback (since it is unchanged in the isolated spinal cord), there may be some kind of feedback that determines the strength of activation, but it is not clear what the sensory input would need to be.

The constancy of tail beat amplitude and wave speed ratio, when the simulations are performed according to the scheme in Fig. 3 and Fig. 4B, indicates that there may be some validity to this scheme, since such constancy is seen in anguilliform swimmers.

The relative timing between activation and curvature is critical for the development of force. The activation wave determines the timing of force development, and the curvature wave the timing of muscle lengthening and shortening. The force developed depends crucially on whether force is developed while the muscle is lengthening or shortening, and by how much (Josephson, 1999). The ratio seen in the swimming lamprey at all speeds, approximately 0.72 (Williams et al., 1989), is similar that found in this study to provide maximum forward speed at a given level of activation. This ratio apparently gives optimal timing between muscle force and extension.

**Fig. 6.**
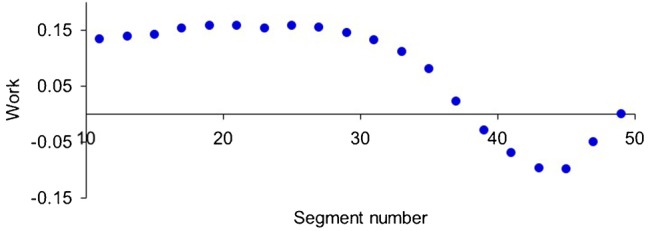
Muscle work against attachments at each segment behind the head. Work calculated as the integral over one cycle of the product of muscle force and muscle shortening. Frequency 4 s^−1^; Activation strength 0.6%.

On the other hand, work done by the muscle decreases monotonically as the ratio of the wave speeds decreases from 1.0, since at the lower ratios more of the muscle is being lengthened during force development. This results in negative work in the tail region, i.e., work is done on the muscle by the sum of the external forces. Hence efficiency in the tail region is sacrificed for swimming speed. The sum of work done at all segments, however, remains positive (Fig. 6), as expected, given that forward swimming occurs.

The values chosen for the parameters of stiffness and damping were within ranges that produced backward-travelling waves of shape and amplitude that resembled those of swimming lampreys. Future studies will explore systematically the effect of changes in these parameters on the quantitative measures of swimming, such as those illustrated in Fig. 5.

A shortcoming of the present study is the use of an oversimplified model for fluid reaction forces. While Taylor's approximation of fluid forces (Taylor, 1952) is very accurate for straight rods in uniform steady flow, it does not capture effects such as vortex shedding that are characteristic of swimming. These effects may be important not only in creating propulsive thrust (Tytell, 2004; Tytell and Lauder, 2004), but the resulting reaction forces on the animal may also influence the speed at which the mechanical wave of curvature travels along its body. Nonetheless, the agreement between the behaviour of the model and that of swimming animals, particular in the ratio between the wave speeds of activation and curvature, indicates that the model captures the essential features of this phenomenon.

## MATERIALS AND METHODS

The computational model of a lamprey body immersed in water acted upon by internal and external forces is based on that published by McMillen, Williams and Holmes (McMillen et al., 2008). The swimmer's body is modeled as an isotropic, inextensible, unshearable, viscoelastic rod that obeys a linear constitutive relation and is subject to hydrodynamic body forces. The equations used in the simulations come from discretizing a continuous rod.

The simulated body consists of a tapered cylinder containing jointed links along the midline, which represent the flexible notochord (Fig. 1A). From these links project perpendicular structures representing the connective tissue septa to which the myotomal swimming muscle attaches (Bowtell and Williams, 1991). Although the body is three-dimensional, it is of constant height. Because anguilliform motion occurs only in a horizontal plane, we use Taylor's model for fluid forces, which well approximates forces for planar motion.

The body is 21 cm long and of uniform density and neutral buoyancy, weighing 15 g. The height of the body is 0.73 cm throughout, with an elliptical cross-section. The maximum width, in the gill region, is 0.73 cm, tapering to 0.05 cm at the tail.

In previous modelling studies, the body width was greatest at the head and tapered uniformly toward the tail (Bowtell and Williams, 1991; Ekeberg, 1993; McMillen et al., 2008). In the present study, the body outline is more realistic (Fig. 1A), as taken from outline data of a lamprey (Leftwich, 2010). In addition, the body consists of a larger number of sections than before (McMillen et al., 2008), in order to minimise the effect of taper (which is ignored in the Taylor approximation). Comparing the resulting forward speed of body models such as that in Fig. 1A which consist of up to 100 sections shows that the speed obtained from a body of 50 sections differs by less than 0.01% from that of 100 sections. Throughout this work, the body had 50 sections, as a compromise between accuracy and computing time.

The force generated by activated swimming muscle segments was calculated using the equations developed by Williams (Williams, 2010), based on data obtained from isolated lamprey muscle (Williams et al., 1998). In brief, the muscle segments are activated by a travelling wave of activation, representing the output of the CPG within the spinal cord. The activation function was a sequence of square waves representing the activation of the swimming muscle segments by their respective motor neurones, as in Bowtell and Williams (Bowtell and Williams, 1991) and McMillen et al. (McMillen et al., 2008), based on the data of Williams et al. (Williams et al., 1989). Although the spinal cord output from each segment is, in actuality, a series of action potentials in a number of neurones, the asynchronous nature of this output and the relative slowness of the resulting muscle depolarisation means that a square wave can be used as a first approximation in the model (Bowtell and Williams, 1991). Based on EMG data from swimming lampreys in a swim-mill (Wallén and Williams, 1984), the square waves occupied 36% of each cycle, alternating on the left and right sides, and travelling down the body at a speed of one body length per cycle at all frequencies (Williams et al., 1989).

An experiment usually consisted of the application of about 8 simulated seconds of activation, as in Fig. 1B, at a prescribed cycle frequency and muscle activation strength. At each hemi-segment, activation produced a rise in muscle force, the value of which was dependent, in the model, on the amount of Ca^2+^ bound to the protein filaments, the length and rate of change of length of the muscle segment, and time-dependent processes (Williams, 2010). At the end of a square wave of activation, the muscle force began to fall, as Ca^2+^ left the protein filaments.

The output of the computation was the simulated forward movement of the animal through the water. Measurements were made of the changing body shape, the forward swimming speed of the centre of mass (situated in segment 18 when at rest) and the speed of the backward-travelling wave of curvature. From the changing body shape the tail-beat amplitude was calculated. These measures were then compared with those observed in other swimming animals (only limited measurements having been made on lampreys).

In the previous study (McMillen et al., 2008) the muscle activation function was a series of square waves starting at time zero. In the current study, this function is multiplied by a *tanh* function which reaches 99% of the maximum in about 3 seconds of simulated time. This has been done in order to allow higher tail beat frequencies than 1 sec^−1^, which otherwise cause slow lateral oscillations of the centre of mass.

The equations of the model are given in the supplementary material Appendix. The only parameters the values of which are not known from nature are those governing the elastic (*v*) and damping 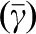 properties of the body tissues (McMillen et al., 2008). Values used were within a wide range which resulted in swimming behaviour similar to that seen in lampreys.

Experiments were performed over a cycle frequency range of 2–6 cycles per second, which is within the range recorded from lampreys in a swim-mill (1.5–7.6 cycles s^−1^; Wallén and Williams, 1984). The range of muscle activation was 0.1% to 20% of maximum isometric strength (Williams et al., 1998; Williams, 2010), which gave rise to changing body shapes closely resembling those seen in swimming lampreys.

## Supplementary Material

Supplementary Material
