# Supplementary material for: Strategies for swimming: explorations of the behaviour of a neuro-musculo-mechanical model of the lamprey
Source: Biol Open. 2015 Feb 6;4(3):253–8. doi: 10.1242/bio.20149621 (PMC4359731; doi:10.1242/bio.20149621)
Supplement: Supplementary Material [file supp_4_3_253__index.html]

Strategies for swimming: explorations of the behaviour of a neuro-musculo-mechanical model of the lamprey — Strategies for swimming: explorations of the behaviour of a neuro-musculo-mechanical model of the lamprey — Supplementary Material 

# Strategies for swimming: explorations of the behaviour of a neuro-musculo-mechanical model of the lamprey

## bio.20149621 Supplementary Material

**Files in this Data Supplement:**

- Supplementary Material - Thelma L. Williams and Tyler McMillen doi: 10.1242/bio.20149621
