# Supplementary material for: Strategies for swimming: explorations of the behaviour of a neuro-musculo-mechanical model of the lamprey
Source: Biol Open. 2015 Feb 6;4(3):253–8. doi: 10.1242/bio.20149621 (PMC4359731; doi:10.1242/bio.20149621)
Supplement: Supplementary Material [file supp_bio.20149621_bio.20149621-s1.pdf]

6 Appendix

6.1 The body model

The model of the lamprey used in this study has been modified from that described in detail in McMillen and Holmes (2006) and McMillen et al. (2008). The model for the muscle forces is that described in Williams (2010).

The swimmer's body is modeled as an isotropic, inextensible, unshearable, viscoelastic rod that obeys a linear constitutive relation and is subject to hydrodynamic body forces. The equations used in the simulations come from discretizing a continuous rod. The discretized equations are identical to those governing a chain of N massless rigid rods each of length h, with mass m_i at each pivot and at both free ends. The pivots are actuated by passive springs, dashpots, and active force generators (See Fig. 4B in McMillen et al. (2008)). The configuration of the ith link is described by its midpoint (x_i, y_i) and the angle φ_i between its centerline and the x-axis. The equations governing the links are

$$m_i\,\ddot{x}_i = hW_{xi} + f_i - f_{i-1}, \tag{1}$$
$$m_i\,\ddot{y}_i = hW_{yi} + g_i - g_{i-1}, \tag{2}$$
$$J_i\,\ddot{\varphi}_i = M_i - M_{i-1} + \frac{h}{2}(g_i + g_{i-1})\cos\varphi_i - \frac{h}{2}(f_i + f_{i-1})\sin\varphi_i, \tag{3}$$

where J_i is the moment of inertia of the ith link, f_i, g_i the components of the contact forces keeping the links connected, and W_{xi}, W_{yi} the body force acting on the ith link. M_i is the moment acting on the ith link. We assume that the cross sections of the rod are elliptical with semi-axes a (height) and b (width).

Actuators generate contractile muscle forces f_{Li} and f_{Ri} on the right and left sides of the body respectively, at a distance $w = b/2$ from the centerline. For small angles, the torque at joint i is given by

$$\begin{aligned} M_i &= [f_{Li}(t) - f_{Ri}(t)]\,w \\ &\quad + \left[2\nu w^2 - \frac{h^2}{4}(f_{Li}(t) + f_{Ri}(t))\right]\left(\frac{\varphi_{i+1} - \varphi_i}{h}\right) + 2\gamma w^2\left(\frac{\dot{\varphi}_{i+1} - \dot{\varphi}_i}{h}\right). \end{aligned} \tag{4}$$

where ν and γ are the stiffness and visco-elastic damping, respectively. In McMillen et al. (2008) we derive the full nonlinear equations for moment acting on the joint. In numerical simulations we found no appreciable difference between the model using the nonlinear equations or the linear constitutive relation in equation (4). Thus, in the present study we assume a linear constitutive relation. The best match of the behaviour of the model with the qualitative behaviour of swimming lamprey is obtained when the damping γ is scaled by cross-sectional area

$$\gamma = ab\,\bar{\gamma} \tag{5}$$

and ν is constant along the length of the body.

In order to relate the parameters in the discrete model to elastic properties of the animal, we note that the curvature κ of the rod is, in the continuum limit, $\kappa = \varphi_s = \lim_{h\to 0}\frac{\varphi_{i+1}-\varphi_i}{h}$. In the absence of external forces, the stiffness EI of a rod is related to the moment M acting on it by $M = EI\kappa$. This means that the stiffness EI in the discrete model of equation (4), is given by

$$EI = 2\nu w^2.$$

Taking the moment of inertia to be $I = \frac{\pi}{4}ab^3$, this means that the Young's modulus is not constant, but varies along the body according to

$$E = \frac{2\nu}{\pi ab} \tag{6}$$

The scaling of E by (6) reflects the fact that the notochord makes up an increasing proportion of the body as the cross sectional area tapers toward the tail. For the values of the parameters used in this model, the Young's modulus varies between 6 and 80 KPa.

The elasticity of the lamprey notochord has not been measured. Amongst the notochords whose elasticity has been measured, the values range from 0.6MPa in sturgeon (Long, 1995) to 4 MPa in hagfish (Long, 2002). Vertebrate muscle has elasticity of approximately 2 kPa (Chen et al, 1996). With such a wide range of values observed, the values chosen for the body of the lamprey in the current study were those that gave rise to swimming resembling that of the lamprey. Future work will explore the dependence of the behaviour on the values chosen.

It is also the case that the viscosity of lamprey tissues has not been measured, but the value chosen had little effect on the outcome (see McMillen et al. (2008)). This too can be explored in further study.

6.2 Approximation of hydrodynamic reaction forces

In swimming the local body forces (W_{xi}, W_{yi}) are due to hydrodynamic reactions that depend on the global velocity field of the fluid relative to the body. To avoid the complexity and computational expense of solving coupled rod and Navier-Stokes equations, we adopt the model of G.I. Taylor (Taylor, 1952) in which $\mathbf{W}$ depends only on the local relative velocity.

Drag forces for smooth cylinders of radius a can be decomposed into normal and tangential components in terms of the normal and tangential velocities $v_\perp$ and $v_\|$ as:

$$F_N = a\rho_f v_\perp^2 + \sqrt{8\rho_f a\mu_f}\, v_\perp^{3/2}, \quad F_T = 2.7\sqrt{2\rho_f a\mu_f |v_\perp|}\, v_\|, \tag{7}$$

where ρ_f is the fluid density and μ_f is the dynamic viscosity. In calculating $\mathbf{W}$, only the height $2a$ of the rod is considered, assuming that fluid reaction forces are equal to those on a cylinder of radius a.

6.3 Muscle activation and force generation

The equations governing force generation by the swimming musculature are taken from a model that can predict the force produced during sinusoidal lengthening and shortening of isolated preparations of lamprey muscle (Williams et al., 1998). For the derivation of these equations, see Williams (2010).

The model incorporates a simple kinetic regime for the release of Ca^{2+} from the sarcoplasmic reticulum (SR), its binding to protein filament sites and subsequent re-sequestering by

the SR. The kinetics of these processes are described by the following equations:

$$\frac{dCa}{dt} = (k_4 Caf - k_3 Ca)(1 - Caf) + \begin{cases} k_1 (C - Ca - Caf), & \text{stimulus on} \\ k_2\, Ca\,(C - S - Ca - Caf), & \text{stimulus off} \end{cases} \tag{8}$$

$$\frac{dCaf}{dt} = -(k_4 Caf - k_3 Ca)(1 - Caf), \tag{9}$$

where Ca and Caf denote the concentrations of free and filament-bound Ca^{2+}, respectively. C and S represent (non-dimensional) total concentrations of Ca^{2+} and SR binding sites, respectively, and $k_1 - k_4$ are rate constants.

The force generated in response to Caf is governed by the following two equations, based on an expansion of Hill's mechanical model of skeletal muscle (Hill, 1949):

$$l_c(t) = L(t) - P(t)/\mu, \tag{10}$$

$$v_c(t) = V(t) - \frac{d}{dt}(P/\mu) \tag{11}$$

where L is the length of the muscle of a segment and l_c the length of its contractile component. V and v_c are the rates of change of L and l_c, respectively. P is the force transferred to the muscle attachments via the series elastic component (SE).

The value of μ, the stiffness of SE, is dependent upon the level of muscle activation, as shown by Josephson (1999), and modelled as follows (Williams, 2010):

$$\mu(t) = \mu_0 + \mu_1\, Caf, \tag{12}$$

where μ_0 is the resting level and μ_1 the constant of proportionality.

The force P_c exerted by the contractile element is described by independent multiplicative factors of its length l_c and velocity v_c,

$$P_c = P_0\, \lambda(l_c)\, \alpha(v_c)\, Caf, \tag{13}$$

where P_0 is the isometric force at optimal length (l_0) and

$$\alpha(v_c) = 1 + \begin{cases} \alpha_m\, v_c & \text{if } v_c < 0 \\ \alpha_p\, v_c & \text{if } v_c \geq 0 \end{cases}, \tag{14}$$

$$\lambda(l_c) = 1 + \lambda_2 (l_c - l_{c0})^2. \tag{15}$$

The quantities α_m, α_p and λ_2 are constants, and $\alpha(v_c)$ is restrained to a maximum value $\alpha_{\max}$.

Work-dependent deactivation (Josephson, 1999) is modeled by the introduction of a variable q, which affects the rate of Ca^{2+} binding and release (Williams, 2010).

$$\frac{dq}{dt} = \begin{cases} -k_{q1} P_c v_c, & v_c < 0 \\ -k_{q2}(q - 1), & v_c \geq 0 \end{cases} \tag{16}$$

The effect of q is to alter the ratio of k_3 and k_4:

$$k_3 = k_{30}/\sqrt{q}$$
$$k_4 = k_{40}\sqrt{q} \tag{17}$$

where k_{30} and k_{40} are constants.

The transfer of force from the CE to the SE is modelled by simple linear kinetics:

$$\frac{dP}{dt} = k_5 \left(P_c - P\right), \tag{18}$$

where k_5 is a constant.

6.4 The integrated model

Muscle dynamics is incorporated into the discretized rod model as follows. The forces P_{Ri} and P_{Li} generated by the right and left muscle segments associated with the ith link are modeled by two sets of the three equations governing the calcium dynamics and muscle forces, with maximal force P_0 scaled by cross-sectional body area at that location. Thus, if the entire body length is actuated, $6(N-1)$ first order ODEs describe the muscle forces in the N-link chain, and with the $3N$ second order ODEs (1-3) they jointly determine the body dynamics.

Symbol	Quantity
Ca	Concentration of free Ca^{2+} in muscle
Caf	Concentration of Ca bound to filaments
CE	Contractile Element
E	Young's modulus of stifffness
F_N	Normal component of drag force
F_T	Tangential component of drag force
f_{Li}	Contractile muscle force on left side
f_{Ri}	Contractile muscle force on right side
h	Length of body segment, L/N
k_3	Rate constant, Ca^{2+} binding to filaments
k_4	Rate constant, Ca^{2+} release from filaments
L	Length of muscle within hemi-segment
l_c	Length of muscle contractile element
M_i	Moment acting on ith link
ODE	Ordinary Differential Equation
P	Force generated by muscle within hemi-segment
P_{Li}	Force generated by left side of segment
P_{Ri}	Force generated by right side of segment
q	Variable governing WDD
SE	Series Elastic
SR	Sarcoplasmic Reticulum
V	Rate of change of L
v_c	Rate of change of l_c
WDD	Work-dependent deactivatiom
W_{xi}, W_{yi}	Body force acting on the ith link
x_i	Horizontal position of segment midpoint
y_i	Vertical position of segment midpoint
$\alpha(v_c)$	Muscle force factor dependent on v_c
$\lambda(l_c)$	Muscle force factor dependent on l_c
μ	Stiffness of SE
v_c	Rate of change of length of l_c
$v_\perp$	Normal velocity of fluid
$v_\parallel$	Perpendicular velocity of fluid
φ_i	Angle between segment centreline and axis

Table 1: Abbreviations and variables used in simulations

Symbol	Quantity	Value	Reference
a	Vertical width of body	0.73cm	(Leftwich, 2010)
b	Horizontal width of body	0.05-0.73cm	(Leftwich, 2010)
C	Total concentration of Ca in muscle	2	(Williams, 2010)
k_1	Rate constant, Ca^{2+} binding in SR	$9sec^{-1}$	(Williams, 2010)
k_2	Rate constant, Ca^{2+} release from SR	$50sec^{-1}$	(Williams, 2010)
k_{30}	Coefficient of variable k_3	$40sec^{-1}$	(Williams, 2010)
k_{40}	Coefficient of variable k_4	$19.4sec^{-1}$	(Williams, 2010)
k_5	Rate constant, transfer CE force to SE	$200sec^{-1}$	(Williams, 2010)
k_{q1}	Rate constant of q increase	$15sec^{-1}$	(Williams, 2010)
k_{q2}	Rate constant of q decrease	$10sec^{-1}$	(Williams, 2010)
L_{is}	Length of muscle segment *in situ*	2.7mm	(Williams, 2010)
L_0	Optimal length of muscle segment	2.94mm	(Williams, 2010)
N	Number of segments to body	50	See text
P_0	Maximal tetanic isometric force	$67kPa/mm^2$	(Williams, 2010)
S	Concentration of Ca^{2+}-binding sites in SR	6	(Williams, 2010)
α_m	Coefficient of $\alpha(v_c)$ for $v_c < 0$	0.8 s/mm	(Williams, 2010)
α_p	Coefficient of $\alpha(v_c)$ for $v_c \geq 0$	2.9 s/mm	(Williams, 2010)
α_{max}	Maximum value for $\alpha(v_c)$	1.8 s/mm	(Williams, 2010)
$\bar{\gamma}$	Constant of viscosity of body tissues	0.2 kg/sec mm	See text
λ_2	Coefficient of $\lambda(l_c)$	$-20mm^2$	(Williams, 2010)
μ_0	Stiffness of SE when $Caf = 0$	1N/m	(Williams, 2010)
μ_1	Gradient of μ against Caf	23N/m	(Williams, 2010)
ν	Stiffness of body tissues	0.5 kg m sec^{-1}	See text
ρ_f	Fluid density of water	$1g/cm^3$	
μ_f	Dynamic viscosiity of water	10^{-3}Pa s	

Table 2: Parameters used in simulations.
